# 

**Published:** 1904-02-27

**Authors:** 


**CADBURY'S COCOA** "A PERFECT FOOD."

**CADBURY'S COCOA** NO DRUGS OR ALKALIES USED.

### THE HOSPITAL

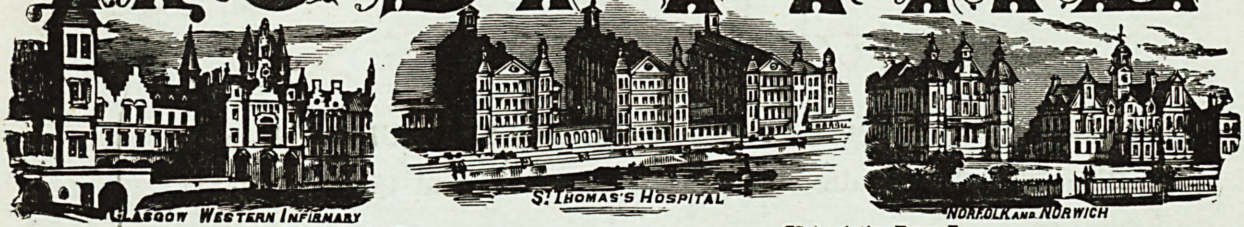

No. 909. Vol. XXXV. [Registered as a Newspaper.] SATURDAY, FEB. 27TH, 1904. [Subscription Terms, see p. 386.] **PRICE THREEPENCE.**

